# Differential effects of *N*-acetylcysteine on retinal degeneration in two mouse models of normal tension glaucoma

**DOI:** 10.1038/s41419-019-1365-z

**Published:** 2019-01-28

**Authors:** Hiroki Sano, Kazuhiko Namekata, Atsuko Kimura, Hiroshi Shitara, Xiaoli Guo, Chikako Harada, Yoshinori Mitamura, Takayuki Harada

**Affiliations:** 1 Visual Research Project, Tokyo Metropolitan Institute of Medical Science, Tokyo, Japan; 2 Department of Ophthalmology, Institute of Biomedical Sciences, Tokushima University Graduate School, Tokushima, Japan; 3 Laboratory for Transgenic Technology, Tokyo Metropolitan Institute of Medical Science, Tokyo, Japan

## Abstract

*N*-acetylcysteine (NAC) is widely used as a mucolytic agent and as an antidote to paracetamol overdose. NAC serves as a precursor of cysteine and stimulates the synthesis of glutathione in neural cells. Suppressing oxidative stress in the retina may be an effective therapeutic strategy for glaucoma, a chronic neurodegenerative disease of the retinal ganglion cells (RGCs) and optic nerves. Here we examined the therapeutic potential of NAC in two mouse models of normal tension glaucoma, in which excitatory amino-acid carrier 1 (*EAAC1*) or glutamate/aspartate transporter (*GLAST*) gene was deleted. EAAC1 is expressed in retinal neurons including RGCs, whereas GLAST is mainly expressed in Müller glial cells. Intraperitoneal administration of NAC prevented RGC degeneration and visual impairment in EAAC1-deficient (knockout; KO) mice, but not in GLAST KO mice. In EAAC1 KO mice, oxidative stress and autophagy were suppressed with increased glutathione levels by NAC treatment. Our findings suggest a possibility that systemic administration of NAC may be available for some types of glaucoma patients.

## Introduction

Glaucoma, an optic neuropathy and a neurodegenerative disease of the eye, is the second leading cause of world blindness^1^. Glaucoma is characterized by slow progressive degeneration of retinal ganglion cells (RGCs) and their axons, which are critical elements for loss of visual function. While this is usually associated with elevated intraocular pressure (IOP), there is a subtype of glaucoma termed normal tension glaucoma (NTG) that present with IOP in a statistically normal range. We previously reported spontaneous mouse models of NTG, and these mice lacked the glutamate transporter gene excitatory amino-acid carrier 1 (EAAC1; *SLC1A1*) or glutamate/aspartate transporter (GLAST; *SLC1A3*)^2,3^. EAAC1 is expressed in retinal neurons including RGCs, while GLAST is mainly expressed in Müller glial cells. EAAC1 transports not only extracellular glutamate but also cysteine, which are important substrates for glutathione (GSH) synthesis, into neural cells^4,5^. GSH, a tripeptide of glutamate, cysteine, and glycine, has a strong protective role against oxidative stress as an antioxidant in the retina^6^. GSH synthesis in Müller glial cells is also considered to be important as GSH may be released from Müller glial cells and may be available to neurons to combat oxidative stress^7^. Several studies have reported that EAAC1-deficient (knockout; KO) mice have decreased neuronal GSH concentrations in the brain^8–10^. In addition, GSH concentration was decreased and lipid hydroperoxides concentration was increased in the retina of EAAC1 and GLAST KO mice, suggesting that NTG-like neurodegeneration may occur at least partly, due to oxidative stress^2,11–15^. Oxidative stress is one of the pathogenic factors for glaucoma and the plasma level of GSH is decreased in primary open angle glaucoma including NTG patients^16,17^, supporting suitability of these mouse models for the basic research of NTG^3,15^.

Autophagy is the major intracellular degradation system and has important roles in the maintenance of cell homeostasis and survival^18,19^. Recent studies indicate a role of autophagy in neurodegenerative diseases including glaucoma^20^. In RGCs, oxidative stress and various forms of stress, such as high IOP, ischemia, and optic nerve transection induce autophagy, and modulation of autophagy processes may result in neuroprotection^21–25^. In addition, mutations in optineurin (OPTN), an adaptor protein that is involved in autophagy, are associated with glaucoma patients, suggesting a role of autophagy in pathogenesis of glaucoma^26,27^. However, there are conflicting results regarding autophagy-induced RGC death. For example, E50K mutated OPTN induced impairment of autophagy that led to cell death in RGC-5 cell lines^28^, and rapamycin, an autophagic inducer, protected RGCs in E50K OPTN overexpressed rats in vivo^29^. On the other hand, M98K OPTN overexpression in RGC-5 cells induced autophagy that led to cell death^30^. In this way, the role of autophagy in glaucomatous RGC death is still controversial.

*N*-acetylcysteine (NAC) is a *N*-acetyl derivative of cysteine and is liposoluble, thus it can permeate across biomembranes, and after entering the cells it can be rapidly hydrolyzed and converted to cysteine^31^. In neurons, the availability of cysteine is the rate-limiting substrate for the synthesis of GSH^32–34^. Usually, cysteine is transported into cells by glutamate transporters, but NAC can provide cysteine to cells without such active transport mechanisms^31,35,36^. Administration of NAC has historically been used as a mucolytic agent in a variety of respiratory illnesses and as an antidote against paracetamol overdose^37–39^. In addition, NAC is recently used for renal protection in contrast-induced nephropathy^40^, as adjuvant therapy in human immunodeficiency virus disease^41,42^, and in the treatment of several psychiatric disorders such as autism, bipolar depression, and schizophrenia^43–45^. In this study, we examined the effect of daily NAC administration on NTG-like retinal degeneration in EAAC1 and GLAST KO mice.

## Results

### NAC is effective for neuroprotection in EAAC1 KO mice

**Fig. 1:**
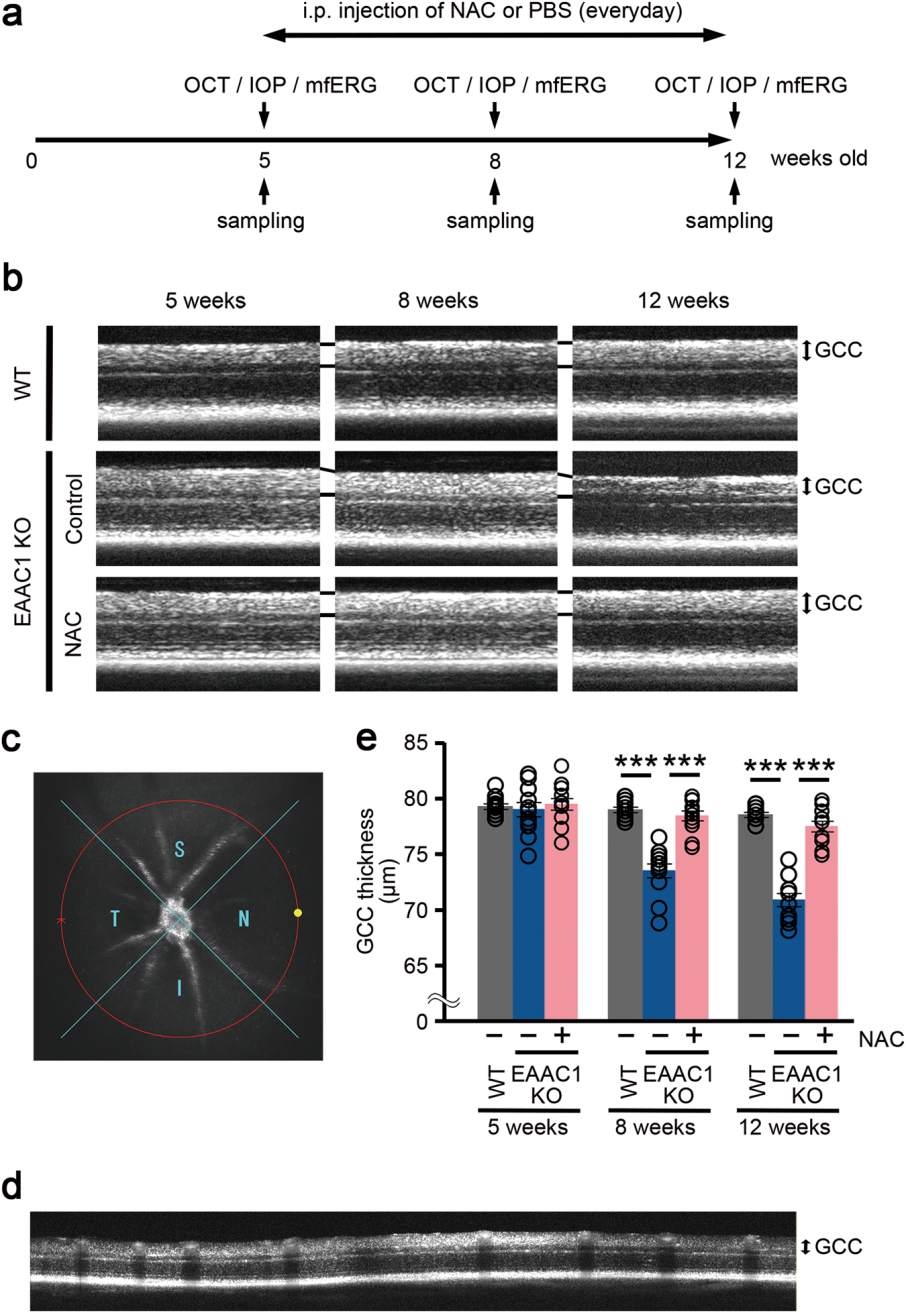
In vivo imaging of the retina in excitatory amino-acid carrier 1 (EAAC1) knockout (KO) mice treated with *N*-acetylcysteine (NAC). **a** Experimental protocols. NAC (200 mg/kg) or phosphate-buffered saline (PBS) was injected intraperitoneally every day from 5 W. The mice were euthanized at 5, 8, and 12 W for further analysis. **b** Optical coherence tomography (OCT) cross-sectional images of retinas at 5, 8, and 12 W. **c** An image of a circle centering around the optic nerve disc. **d** An OCT circular scan image captured from **c**. **e** Longitudinal evaluation of the ganglion cell complex (GCC) thickness by a circular scan. The data are presented as means ± S.E.M. *n* = 12 eyes per group. ***P < 0.001

**Fig. 2:**
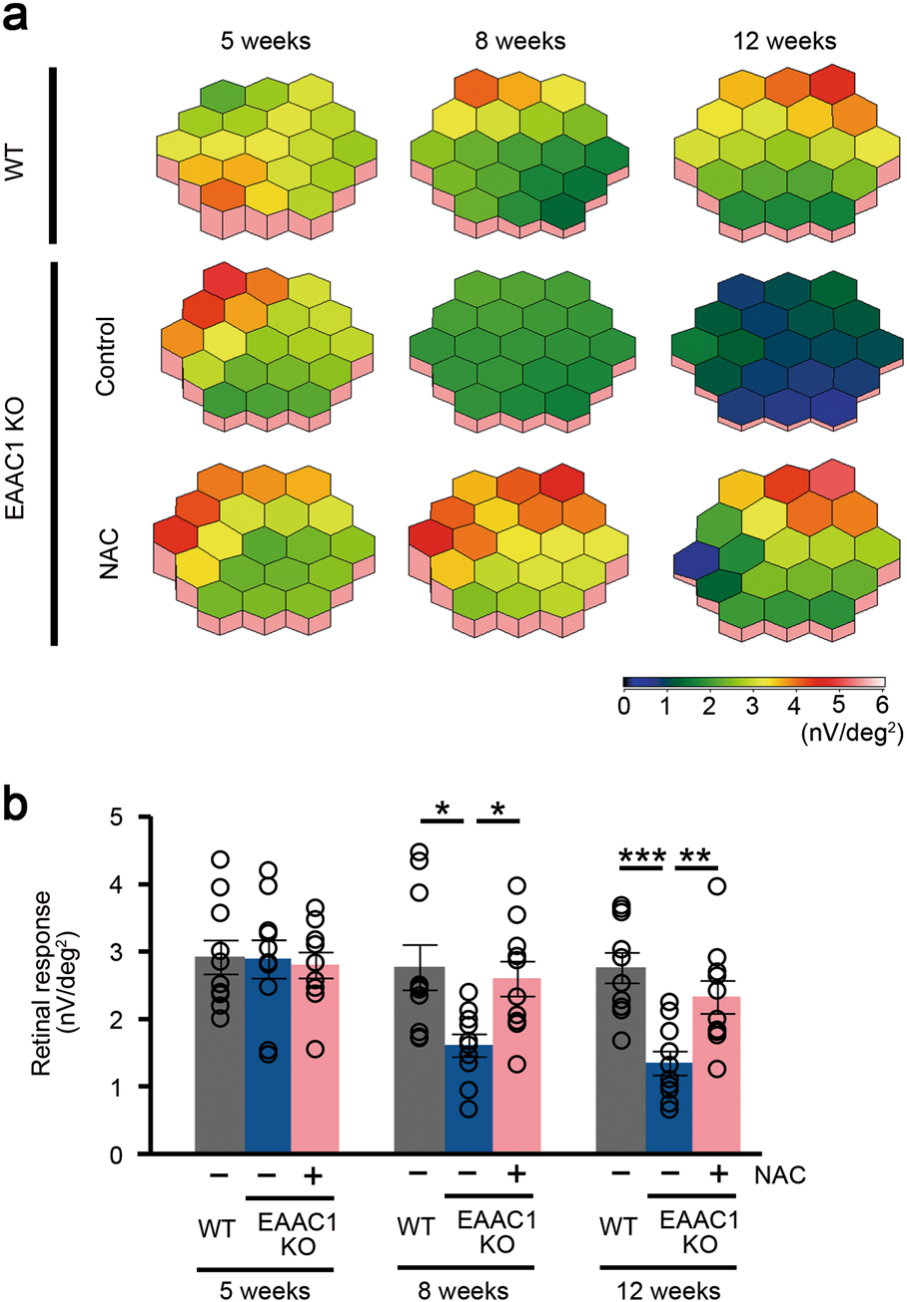
Effects of *N*-acetylcysteine (NAC) on retinal responses examined using multifocal electroretinogram in excitatory amino-acid carrier 1 (EAAC1) knockout (KO) mice. **a** Averaged retinal responses demonstrated using three-dimensional plots at 5, 8, and 12 W. **b** Quantitative analyses of the retinal response amplitude. The data are presented as means ± S.E.M. *n* = 10 eyes per group. *P < 0.05, **P < 0.01, ***P < 0.001

We administered NAC or phosphate-buffered saline (PBS; as a control) intraperitoneally every day to EAAC1 KO mice from 5 weeks of age (5 W) to 8 or 12 W to examine whether NAC can prevent the NTG-like phenotypes in EAAC1 KO mice (Fig. 1a). First, we investigated the thickness of ganglion cell complex (GCC) using spectral domain optical coherence tomography (SD-OCT). GCC, a combination of nerve fiber layer, ganglion cell layer (GCL), and inner plexiform layer, is useful for evaluation of structural changes observed in glaucoma^46–48^. SD-OCT acquires cross-sectional tomographic images of the retina noninvasively, allowing observation of the retinal morphology in living subjects. We found that the GCC thickness was reduced in control mice, but it was almost unchanged in NAC-treated EAAC1 KO mice (Fig. 1b). For quantitative analysis, GCC was measured by circular-scanning around the optic nerve disc (Fig. 1c), and the average GCC thickness was calculated from obtained images (Fig. 1d). The GCC thickness at 8 and 12 W was significantly greater in NAC-treated EAAC1 KO mice than in control mice (Fig. 1e), suggesting that NAC suppressed retinal degeneration. In order to determine if the neuroprotective effect by NAC treatment in EAAC1 KO mice reflects functional aspects, we investigated retinal function using multifocal electroretinogram (mfERG). The second-order kernel, which is impaired in patients with glaucoma, was analyzed as previously reported^2,11,49,50^. The response topography demonstrated that the average retinal responses were impaired in all visual fields in EAAC1 KO mice, but NAC treatment prevented the decline in retinal function (Fig. 2). These results show that the neuroprotective effects of NAC are functionally important.

### NAC is ineffective for neuroprotection in GLAST KO mice

**Fig. 3:**
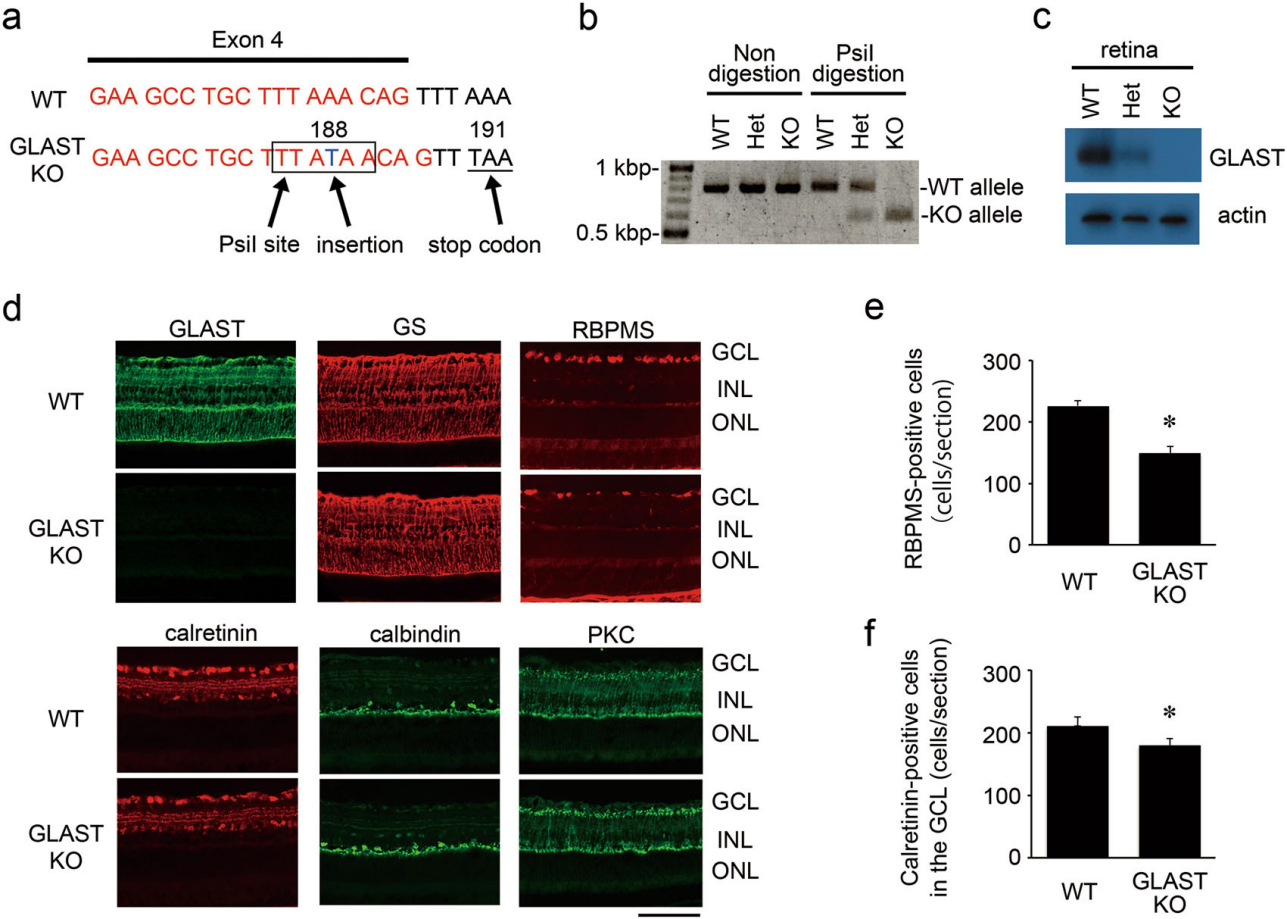
Retinal ganglion cell degeneration in glutamate/aspartate transporter (GLAST) knockout (KO) mice. **a** Genomic DNA sequence of the *GLAST* gene. Thymidine (T), indicated in blue, was inserted in codon 188, and a stop codon was created at codon 191. Exon 4 is indicated in red. The recognition site of the restriction enzyme PsiI is indicated as a square. **b** PCR genotyping of the *GLAST* gene. PCR products from tail DNA were digested with PsiI. The GLAST KO allele, but not the wild-type (WT) allele, was digested with PsiI. **c** Immunoblot analysis of GLAST. The equal amount of retinal protein lysates were resolved by SDS-polyacrylamide gel electrophoresis and assessed by immunoblot analysis with anti-GLAST and anti-actin antibodies. **d** Immunostaining of the retina of WT and GLAST KO mice at 12 W using cell-type-specific markers. Scale bar: 100 µm. GCL ganglion cell layer, INL inner nuclear layer, ONL outer nuclear layer. **e**, f Quantitative analyses of the RNA-binding protein with multiple splicing (RBPMS)-positive cells (**e**) and calretinin-positive cells in the GCL (**f**). The data are presented as means ± S.E.M. *P < 0.01. *n* = 6 eyes per group

In the present study, we generated a new GLAST KO mouse line using CRISPR/Cas9 technology (Fig. 3a) and confirmed the loss of the *GLAST* gene by PCR analysis (Fig. 3b). Immunoblot and immunohistochemical analyses confirmed the absence of the GLAST protein, whereas a stable expression pattern of glutamine synthetase (GS; a marker of Müller glial cells) was observed in GLAST KO mice (Fig. 3c, d). We next examined the GLAST KO mouse retina using an antibody against RNA-binding protein with multiple splicing (RBPMS), a selective RGC marker^51^. Consistent with our previous reports^2,3^, the RBPMS-positive RGC number was decreased in GLAST KO mice compared with wild-type (WT) mice at 12 W (Fig. 3d, e). In addition, we examined the expression of calretinin (a marker of RGCs and amacrine cells) and found calretinin-positive cells are also decreased in the GCL in GLAST KO mice (Fig. 3d, f). To examine the effects of GLAST on other retinal cell types, we carried out immunohistochemistry with calbindin (a marker of horizontal cells) or protein kinase C (PKC; a marker of bipolar cells)^52,53^, but we could detect no differences in their expression patterns between WT and GLAST KO mice (Fig. 3d).

**Fig. 4:**
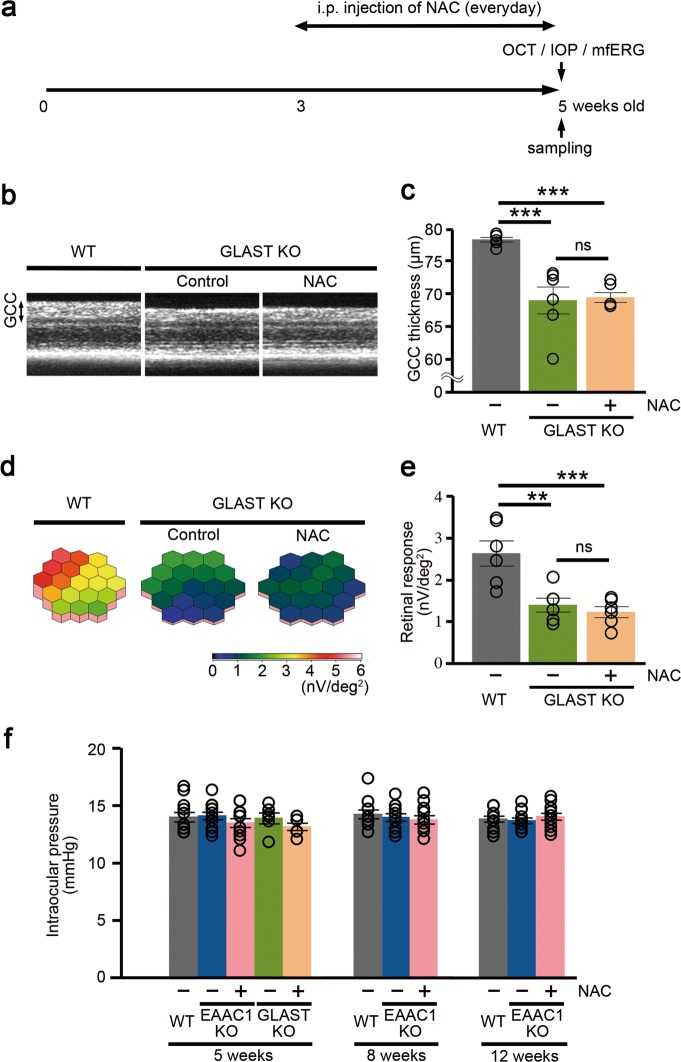
Effects of *N*-acetylcysteine (NAC) on retinal degeneration in glutamate/aspartate transporter (GLAST) knockout (KO) mice. **a** Experimental protocols. NAC (200 mg/kg) was injected intraperitoneally every day from 3 W. The mice were euthanized at 5 W. **b** Optical coherance tomography (OCT) cross-sectional images of retinas at 5 W. **c** Longitudinal evaluation of the ganglion cell complex (GCC) thickness by a circular scan. *n* = 6 eyes per group. **d** Averaged retinal responses demonstrated using three-dimensional plots at 5 W. **e** Quantitative analyses of the retinal response amplitude. *n* = 6 eyes per group. **f** Effect on intraperitoneal administration of NAC in intraocular pressure. *n* = 12 eyes (wild-type (WT) and excitatory amino-acid carrier 1 (EAAC1) KO mice) and 6 eyes (GLAST KO mice). The data are presented as means ± S.E.M. **P < 0.01, ***P < 0.001

To examine whether NAC has similar neuroprotective effects in GLAST KO mice as shown in EAAC1 KO mice, we administrated NAC intraperitoneally every day to GLAST KO mice from 3 to 5 W (Fig. 4a). We investigated the thickness of the GCC using SD-OCT in NAC-treated GLAST KO mice, but the GCC thickness was decreased, similar to control mice (Fig. 4b, c). We then investigated retinal function using mfERG, but NAC treatment did not ameliorate the decline in retinal function in GLAST KO mice compared with controls (Fig. 4d, e). These results show intraperitoneal administration of NAC does not prevent retinal degeneration in GLAST KO mice.

We also investigated the effects of NAC on IOP. We have already reported that the IOP in EAAC1 and GLAST KO mice were not significantly increased compared with WT mice^2,11,12,14,53,54^. The IOP values of NAC-treated mice were not significantly altered compared with the control mice (Fig. 4f), indicating that the neuroprotective effects of NAC in EAAC1 KO mice are not mediated via reduction of IOP.

### NAC protects RGCs in EAAC1 KO mice

**Fig. 5:**
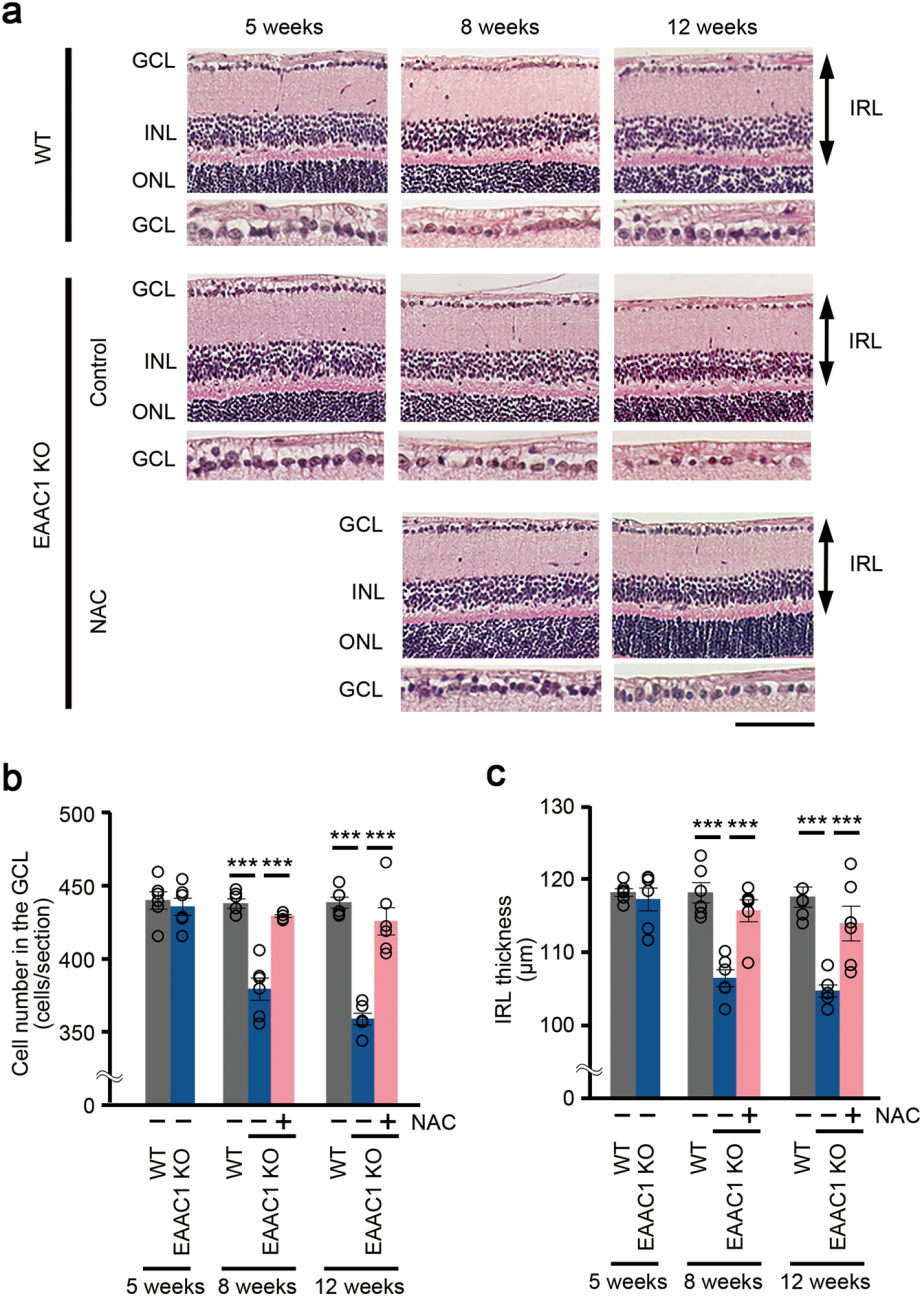
Effects of *N*-acetylcysteine (NAC) on retinal degeneration in excitatory amino-acid carrier 1 (EAAC1) knockout (KO) mice. **a** Hematoxylin and eosin staining of retinal sections. Scale bar: 100 and 50 μm in the upper and immediately lower panels, respectively. IRL inner retinal layer. **b**, **c** Quantitative analyses of the cell number in the GCL (**b**) and IRL thickness (**c**). The data are presented as means ± S.E.M. *n* = 6 eyes per group. ***P < 0.001

**Fig. 6:**
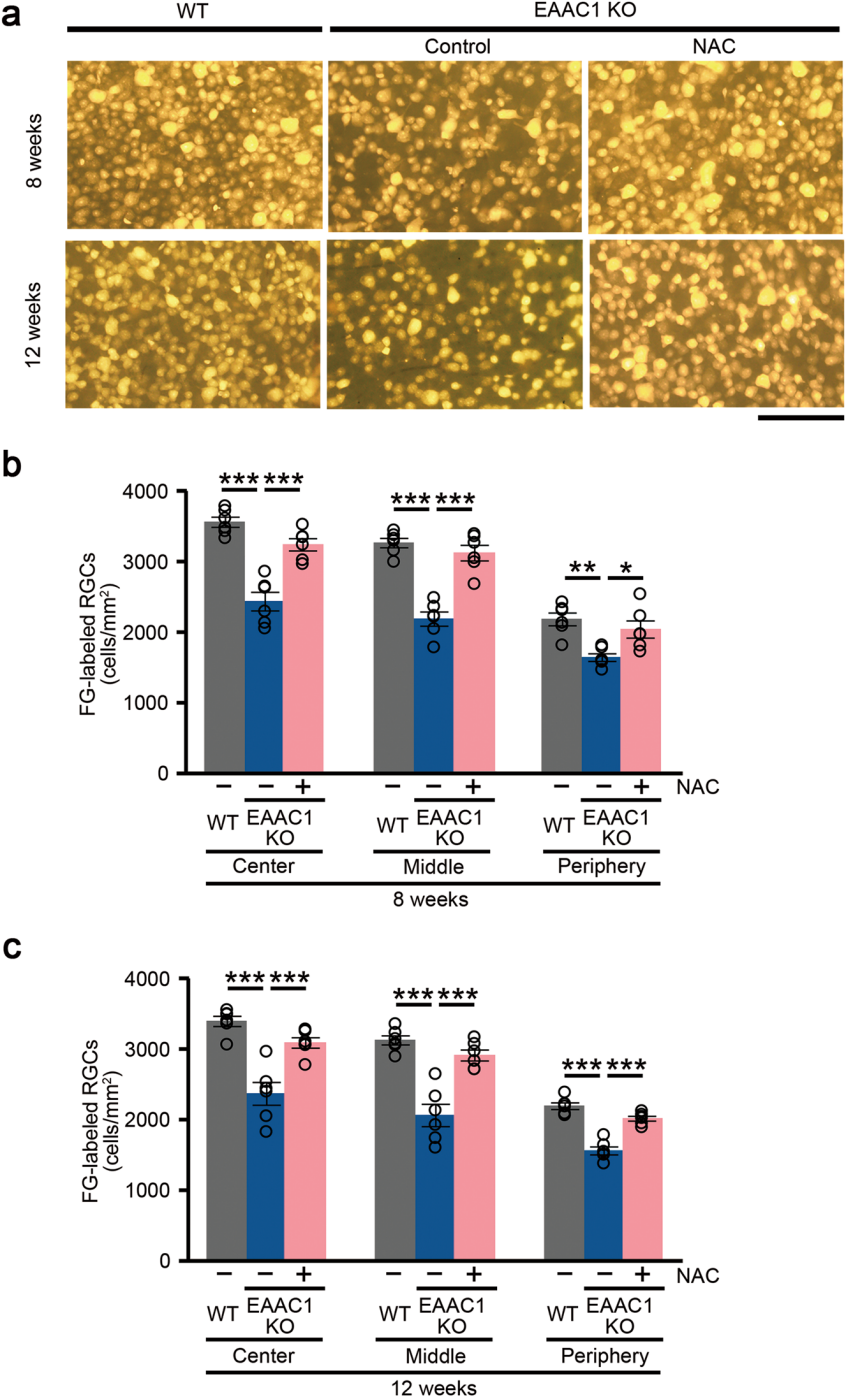
Effects of *N*-acetylcysteine (NAC) on retinal ganglion cell (RGC) degeneration in excitatory amino-acid carrier 1 (EAAC1) knockout (KO) mice. **a** Representative images of retrograde-labeled RGCs at 8 and 12 W. Scale bar: 100 μm. **b**, **c** Quantitative analyses of **a** at 8 W (**b**) and at 12 W (**c**). The data are presented as means ± S.E.M. *n* = 6 eyes per group. *P < 0.05, **P < 0.01, *** P < 0.001

We then examined histopathology of the retina in EAAC1 KO mice. We previously reported that the cell number in the GCL was significantly lower and the thickness of the inner retinal layer (IRL) was significantly reduced in EAAC1 KO mice compared with WT mice at 8 and 12 W^2,11,12,14,53,54^, which is consistent with the decreased GCC thickness detected by SD-OCT. We found that the number of surviving neurons in the GCL was significantly greater in NAC-treated EAAC1 KO mice compared with control mice at 8 and 12 W (Fig. 5a, b). In addition, NAC treatment prevented the thinning of the IRL (Fig. 5a, c). Because the GCL of rodent retinas contains both RGCs and displaced amacrine cells^55^, we next specifically labeled RGCs by retrograde labeling with Fluoro-Gold (FG) to determine the effects of NAC on RGC number in EAAC1 KO mice (Fig. 6a). Consistent with the trends observed in the number of cells in the GCL, the RGC number in NAC-treated EAAC1 KO mice was significantly higher than in control mice all across the retina at 8 and 12 W (Fig. 6b, c). Taken together, these results show that NAC treatment protects RGCs from NTG-like neurodegeneration.

### NAC suppresses the oxidative stress level in EAAC1 KO mice, but not in GLAST KO mice

**Fig. 7:**
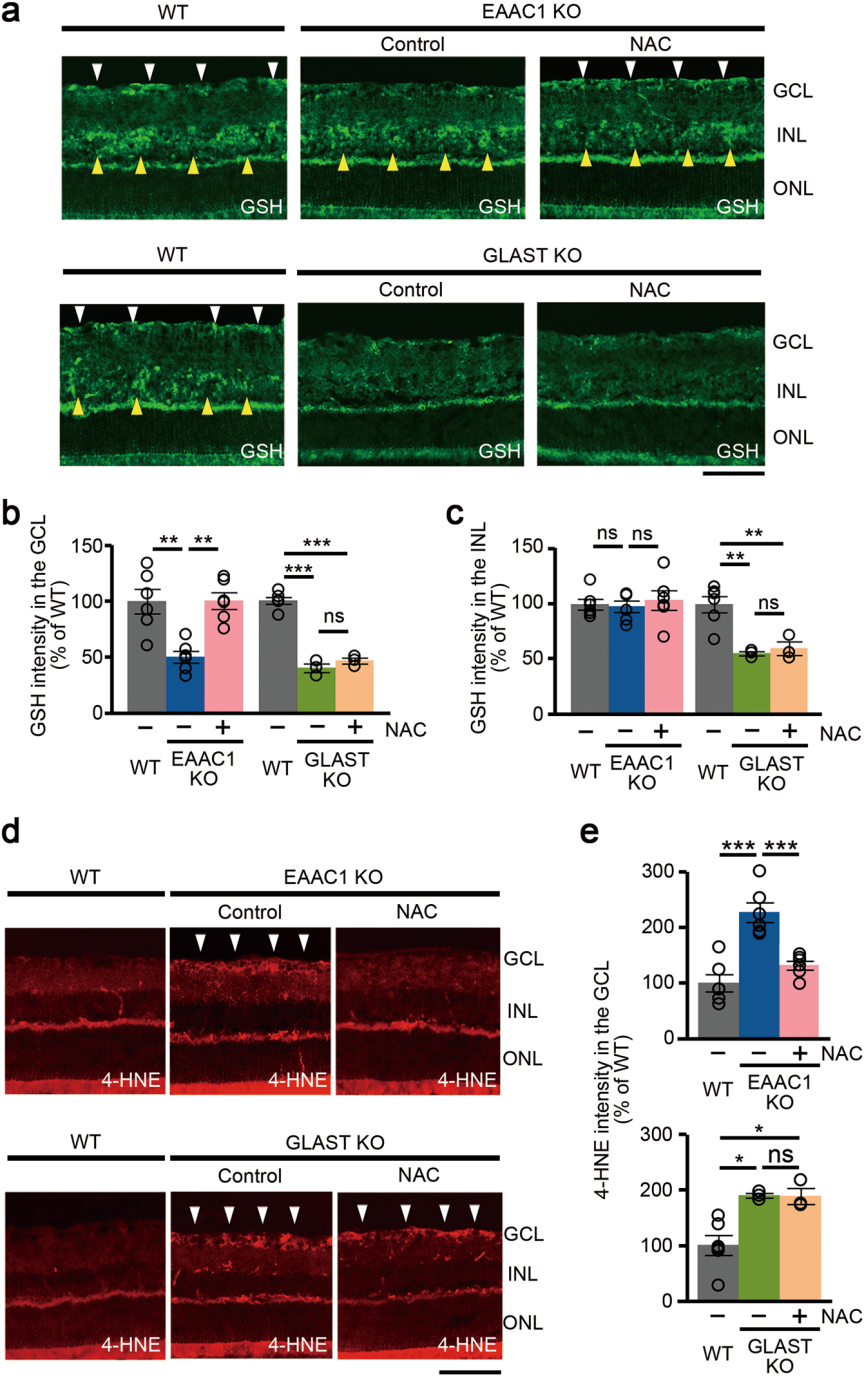
Differential effects of *N*-acetylcysteine (NAC) on oxidative stress levels in excitatory amino-acid carrier 1 (EAAC1) and glutamate/aspartate transporter (GLAST) knockout (KO) mice. **a** Representative images of glutathione (GSH) in the retina. Arrowheads show the high-intensity area in the ganglion cell layer (GCL; white) and inner nuclear layer (INL; yellow). Scale bar: 100 μm. **b**, **c** Quantitative analyses of **a** in the GCL (**b**) and INL (**c**). Data are normalized to the GSH intensity in control wild-type (WT) mice (100%). **d** Representative images of 4-hydroxy-2-nonenal (4-HNE) in the retina. Arrowheads show the high-intensity area in the GCL. Scale bar: 100μm. **e** Quantitative analyses of **d**. Data are normalized to the 4-HNE intensity at the GCL in control WT mice (100%). The data are presented as means ± S.E.M. *n* = 6 eyes (WT and EAAC1 KO mice) and 3 eyes (GLAST KO mice). *P < 0.05, **P < 0.01, ***P < 0.001

We next examined the antioxidant effects of NAC in EAAC1 and GLAST KO mice. Since GSH is the major redox buffer in the retina and decreased GSH levels are found in glaucoma patients^16,17^, we examined GSH expression levels in EAAC1 and GLAST KO mouse retinas. GSH intensity in the GCL, but not inner nuclear layer (INL), of EAAC1 KO mice was significantly lower than that of WT, and NAC treatment suppressed the GSH reduction in the GCL (Fig. 7a–c). In GLAST KO mice, GSH reduction was observed in both the GCL and INL, and NAC had no effects on GSH expression levels.

We then investigated if NAC treatment normalizes oxidative stress levels in EAAC1 and GLAST KO mice. For this purpose, we utilized 4-hydroxy-2-nonenal (4-HNE), which is one of the major products of lipid peroxidation and can be used as a marker for oxidative stress^11–13^. A significant increase of 4-HNE intensity were observed mainly in the GCL of EAAC1 and GLAST KO mice (Fig. 7d). NAC treatment attenuated 4-HNE expression levels in EAAC1 KO mice, but not in GLAST KO mice (Fig. 7e). These results suggest that NAC prevents retinal degeneration in EAAC1 KO mice, but not in GLAST KO mice, at least partly by increasing the GSH levels and suppressing the oxidative stress levels in the retina.

### NAC reduces the microtubule-associated protein 1 light chain 3 beta level in EAAC1 KO mice, but not in GLAST KO mice

**Fig. 8:**
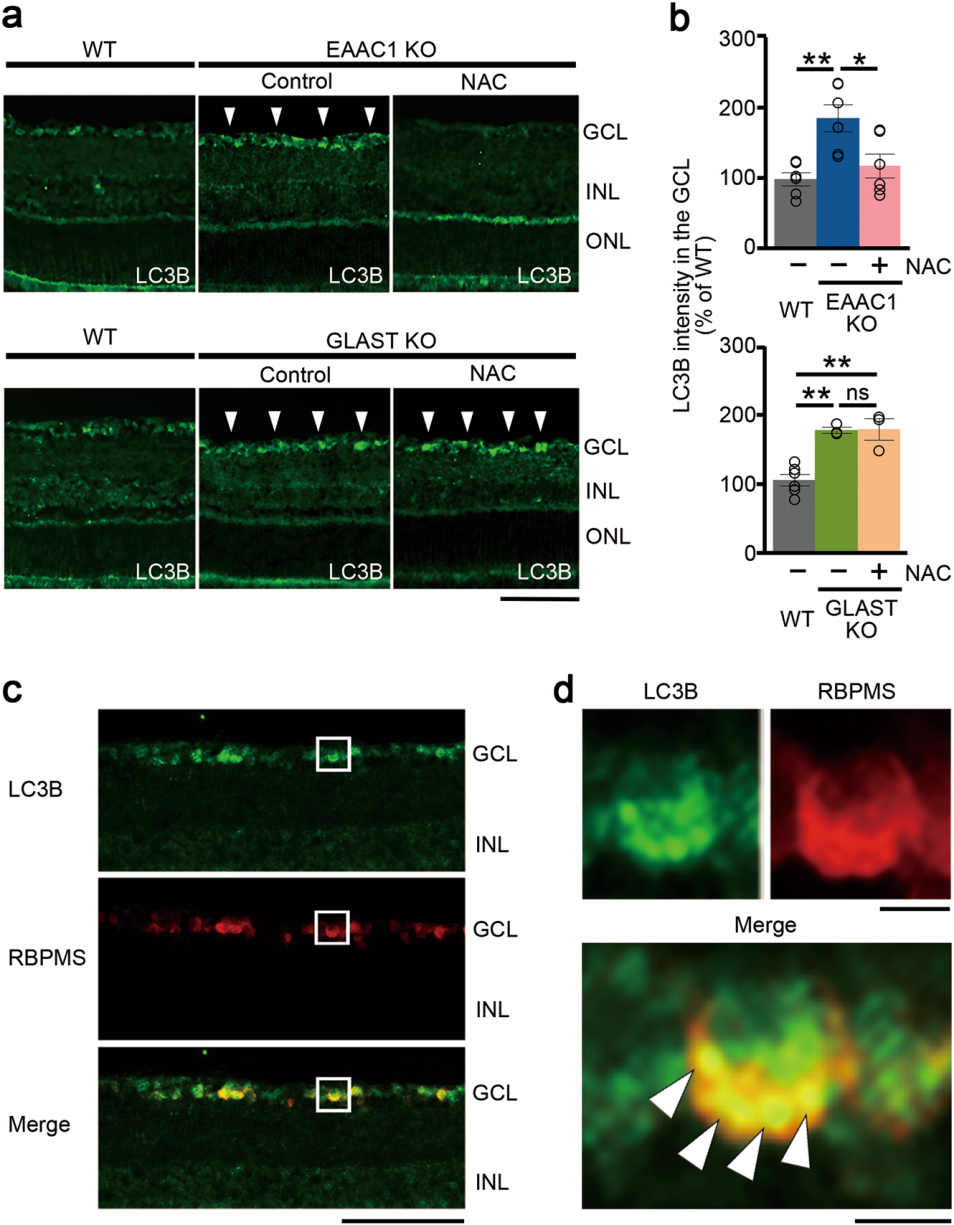
Differential effects of *N*-acetylcysteine (NAC) on autophagy in excitatory amino-acid carrier 1 (EAAC1) and glutamate/aspartate transporter (GLAST) knockout (KO) mice. **a** Representative images of microtubule-associated protein 1 light chain 3 beta (LC3B) in the retina. Arrowheads show the high-intensity area in the ganglion cell layer (GCL). Scale bar: 100μm. **b** Quantitative analyses of **a**. Data are normalized to the LC3B intensity at the GCL in control wild-type (WT) mice (100%). The data are presented as means ± S.E.M. *n* = 6 eyes (WT and EAAC1 KO mice) and 3 eyes (GLAST KO mice). *P < 0.05, **P < 0.01. **c** Representative images of LC3B (green) and RNA-binding protein with multiple splicing (RBPMS; red) in the EAAC1 KO mouse retina. Scale bar: 100 μm. **d** Magnified images of the square areas in **c**. Arrowheads show punctate staining of LC3B in RGCs. Scale bars: 5 μm

Several studies have indicated a role of autophagy in glaucoma^22–25^. Therefore, we finally examined the effect of NAC on autophagy in EAAC1 and GLAST KO mice. For this purpose, we used an antibody against microtubule-associated protein 1 light chain 3 beta (LC3B), which is one of the major biochemical markers of autophagy^56^. A significant increase in LC3B intensity was observed mainly in the GCL of EAAC1 and GLAST KO mice, and NAC treatment attenuated LC3B expression levels in EAAC1 KO mice, but not in GLAST KO mice (Fig. 8a, b). To further investigate whether LC3B is expressed in RGCs, we performed co-localization studies using antibodies against LC3B and RBPMS, in EAAC1 KO mice. We found that some LC3B-positive cells are double-labeled with RBPMS in EAAC1 KO mice (Fig. 8c, d). These results suggest that autophagy is activated in RGCs of EAAC1 and GLAST KO mice, and NAC treatment suppresses autophagy in EAAC1 KO mice, but not in GLAST KO mice.

## Discussion

Our principal aim of the present study was to examine the neuroprotective effect of daily NAC administration on retinal degeneration in two mouse models of NTG. Using histological and functional assessments, we demonstrated that NAC exerts neuroprotective effects in EAAC1 KO mice without affecting IOP. We also demonstrated that NAC increases the retinal GSH levels and reduces 4-HNE, which represent decreased oxidative stress levels, in the GCL of EAAC1 KO mice. In addition, we demonstrated that NAC suppresses autophagy in RGCs of EAAC1 KO mice. Interestingly, NAC did not affect GSH levels or suppress retinal degeneration in GLAST KO mice. GSH has a protective role against oxidative stress as an available antioxidant in various tissues including retina, and the reduction of GSH levels is found in several neurodegenerative disorders, such as Parkinson’s disease and schizophrenia, associated with oxidative stress^6,57,58^. In glaucoma patients, the plasma levels of GSH and total antioxidant capacity are decreased^16,17,59^. For this reason, targeting oxidative stress in the retina may be a novel therapeutic strategy for glaucoma^15^. Previous experiments by our group have shown that retinal degeneration in EAAC1 KO mice was suppressed by administration of edaravone, a free radical scavenger, and spermidine, a polyamine with an antioxidant property^11,13^. Consistently, NAC, an antioxidant and a precursor of the synthesis of GSH, prevented RGC degeneration in EAAC1 KO mice. These findings suggest that oxidative stress plays a major role in the NTG-like RGC death in EAAC1 KO mice.

NAC is also an antioxidant itself as a source of sulfhydryl groups in cells and scavenger of free radicals as it interacts with reactive oxygen species such as OH and H_2_O_2_^60^. However, the direct effect is believed to be negligible when compared to its indirect, pro-GSH activity^61–63^. Several studies have reported that NAC can replete neuronal GSH in the brains of EAAC1 KO mice^8–10^. In addition, NAC increases the GSH levels and reduces the upregulation of 4-HNE in the retina resulting from oxidative stress by light exposure^6^. The levels of 4-HNE are remarkably elevated under conditions of oxidative stress and it is considered as one of the reliable biomarker of oxidative stress^64^. The present study demonstrated that NAC suppresses the retinal expression levels of 4-HNE in EAAC1 KO mice. Taken in this light, it is reasonable to support that NAC prevents retinal degeneration in EAAC1 KO mice by reducing the oxidative stress levels in the retina by increasing the GSH levels.

On the other hand, we found that NAC did not increase GSH levels or prevent retinal degeneration in GLAST KO mice. What caused the different outcome between the two NTG model mice? GLAST has essential roles in keeping the extracellular glutamate concentration below the neurotoxic level by transporting glutamate into Müller glial cells, and also in synthesis of GSH in Müller glial cells^2,65,66^. Interestingly, the rate-limiting substrate for GSH synthesis in Müller glial cells is glutamate, and not cysteine^66–68^. This suggests that although plenty of cysteine was available through NAC, glutamate was lacking from Müller glial cells in GLAST KO mice and therefore, GSH synthesis was not promoted. It is also possible that glutamate neurotoxicity may play a bigger role than oxidative stress in RGC death observed in GLAST KO mice. In this case, it raises an important point that although these two NTG models both lack glutamate transporters, their cell-type specificity makes the major contributing factor for RGC death in these two models different, highlighting independent values of these two models. To further determine the role of GSH in GLAST KO mice, it would be interesting to test whether GSH ethyl ester, which restores mitochondrial GSH in vivo^69^, can rescue RGC degeneration in GLAST KO mice. This could help answering whether glutamate in the GLAST KO model was indeed the rate-limiting substrate from GSH synthesis or if neurodegeneration was mainly due to glutamate neurotoxicity.

We found that autophagy is activated in RGCs, and possibly also in displaced amacrine cells, in EAAC1 and GLAST KO mice, and NAC treatment suppresses autophagy only in EAAC1 KO mice. Several studies have reported that autophagy prevents RGC death in glaucoma animal models such as optic nerve transection and IOP elevation^24,25^. In contrast, inhibition of autophagy by intravitreal injection of 3-methyladenine, a potent inhibitor of autophagosome maturation, was neuroprotective in ischemia and chronic IOP elevation rat models^22,23^. Interestingly, with progressive increase in IOP, autophagy is initially activated in the dendrites of RGCs and protects cells, but its subsequent activation in the cytoplasm disrupts homeostasis and induces cell death^21,22,70–72^. Therefore, it is possible that the timing and the location of autophagy may be important for its pro- or anti-cell death effects. In the present study, it is presumed that autophagy is induced by increased oxidative stress in EAAC1 and GLAST KO mice, and NAC suppressed autophagy and promoted RGC protection by reducing oxidative stress. However, whether the modulation of autophagy is directly related to RGC survival in our study requires further investigation.

We have used EAAC1 and GLAST KO mice as an animal model of NTG in this study. As with any animal models of disease, some aspects of biological process that occur in glaucoma patients are not reflected in these animal models^3^. For example, in humans, it typically takes years to develop visual field loss, and treatment typically starts long time after the onset of disease, whereas in these mice, neurodegeneration starts at 5 W (EAAC1 KO mice) or 3 W (GLAST KO mice) and the treatment was started at the onset of neurodegeneration. In addition, the RGC deaths in these mice are distributed across the whole retina, rather than in specific regions as seen in human glaucoma. However, these models are important because they mimic pathology of NTG, including RGC loss, optic nerve atrophy, and visual impairment with normal IOP^2,3^. One may speculate that RGC loss in these NTG models are due to degeneration in the brain, leading to loss of neural connections between the visual center in the brain and the retina. However, GLAST is mainly expressed in the cerebellum and no brain degeneration is reported in GLAST KO mice^73^. In addition, EAAC1 is mainly localized to the forebrain including the hippocampus and no neurodegeneration was observed during a period of over 12 months in EAAC1 KO mice^74^. In some studies, brain atrophy and behavioral abnormalities were observed in EAAC1 KO mice at 11 months old, but not at young ages^8,9^, although such degeneration has not been universally reported^75^. In any case, we examined EAAC1 KO mice before 3 months old when no neurodegeneration was observed in the brain. Therefore, these animal models are useful for investigating the novel therapeutic approaches for NTG, easily and speedily^2,3,11–15,49,53,54^.

NAC is relatively safe and well tolerated, with occasional mild side effects^61^. In clinical practice, systemic administration (orally or intravenously) of NAC is used as an antidote of paracetamol overdose; 140 mg/kg as the loading dose, and then 70 mg/kg every 4 h for 3 days^76,77^. The dose of NAC (200 mg/kg) used in our present study is similar to this clinical dose. It follows from these findings that the systemic administration of NAC may be available for glaucoma therapy. However, we should be careful of the side effects of long-term administration of NAC, as some studies indicate that it may accelerate lung cancer and melanoma metastasis in mice^78,79^.

In conclusion, the results of this study demonstrate that intraperitoneal administration of NAC prevents NTG-like retinal degeneration by increasing the GSH levels and suppressing oxidative stress and autophagy in EAAC1 KO mice, but not in GLAST KO mice. Our findings raise an intriguing possibility that systemic administration of NAC may be effective for a type of glaucoma that is associated with increased oxidative stress.

## Materials and methods

### Mice

Procedures using laboratory animals were followed in accordance with the Tokyo Metropolitan Institute of Medical Science Guidelines for the Care and Use of Animals (Approval numbers: 14046 and 16082). All efforts were made to minimize animal suffering and the number of animals used. Experiments were performed using EAAC1 KO mice (Miltenyi Biotec GmbH, Bergisch Gladbach, Germany)^2,11–14,53^ and GLAST KO mice on a C57BL6 background. GLAST KO mice were generated using CRISPR/Cas9 technology. The Cas9/sgRNA target sequence was designed from the GLAST genomic sequence (NCBI Reference Sequence: NC_000081.6) and was followed by the underlined PAM sequences: 5′-GGTAGAAGCCTGCTTTAAACAGG-3′. One nucleotide was inserted at exon 4, resulting in creation of a stop codon at codon 191 (Fig. 3a). Genotyping was performed by PsiI digestion of the PCR product. PCR was performed using following primer sets; 5′-GTGCGCGTGTGAATAATATGCAGGTGCCTGAAGAAGCTAG-3′ and 5′-CCTAGCAAGAAGCACACTACATCTTCAAAAGGAACTTTGA-3′. To genotype mice carrying the mutation in GLAST exon 4, PCR products (824 bp) were digested with PsiI, which gives two fragments of 604 and 220 bp in the mutant gene. The PCR product from WT is not digested by PsiI. All mice were housed under a 12-h light-dark cycle (light on at 8:00, light off at 20:00 pm) and were allowed free access to food and drinking water.

### Drug administration

Mice were treated with intraperitoneal administration of NAC (200 mg/kg; Nacalai Tesque, Kyoto, Japan) dissolved in PBS every day from 5 to 8 or 12 W (EAAC1 KO mice), or from 3 to 5 W (GLAST KO mice). The dose of NAC was determined based on previous studies that showed beneficial effect of NAC in the eye^6,80,81^. PBS was administered as a control.

### Imaging acquisition of SD-OCT

SD-OCT (RS-3000; Nidek, Aichi, Japan) imaging was performed at 5, 8, and 12 W. Before examination, mice were anesthetized with an intraperitoneal injection of sodium pentobarbital (64.8 mg/kg) and the pupils were dilated with a mixed solution of 0.5% phenylephrine and 0.5% tropicamide. The eyes were fitted with polymethyl methacrylate contact lenses optimized for mice (UNICON, Osaka, Japan) to prevent drying up of the cornea and anesthesia-induced cataract progression. To focus on the mouse retina, optical qualities of the mouse eye were adjusted by using a 60-D fundus lens (Ocular Instruments, Bellevue, WA, USA) that is placed in front of the objective lens of the OCT. All the line scan images were obtained at location of three-disc diameter distance from the optic nerve disc, and the average thickness of the GCC (from the inner limiting membrane to the outer boundary of the inner plexiform layer) was measured by circular-scanning around the optic nerve disc^11–14,53,82^.

### Multifocal electroretinogram

The mfERGs were recorded using a VERIS 6.0 system (Electro-Diagnostic Imaging, Redwood City, CA, USA) at 5, 8, and 12 W. Before examination, mice were anesthetized with an intraperitoneal injection of sodium pentobarbital (87.5 mg/kg) and the pupils were dilated with a mixed solution of 0.5% phenylephrine and 0.5% tropicamide. The visual stimulus consisted of seven hexagonal areas scaled with eccentricity. The stimulus array was displayed on a high-resolution black and white monitor driven at a frame rate of 100 Hz. The second-order kernel was analyzed as previously reported^2,11,49,50^.

### IOP measurement

IOP was measured by a commercial rebound tonometer (TonoLab; Colonial Medical Supply, Franconia, NH, USA) at 5, 8, and 12 W as we previously described^11,14,49,53^. Before examination, mice were anesthetized with an intraperitoneal injection of sodium pentobarbital (64.8 mg/kg). The tonometer was placed in front of the eye, and IOP was measured with the tip of the probe touching the central cornea vertically. To minimize variation, the data were collected between 15:00 and 18:00, 4–6 min after injection of the anesthetic, during which time IOP plateaus.

### Histologic and morphometric studies

Mice were perfused with ice-cold PBS, followed by Zamboni’s fixative (0.1 M phosphate buffer containing 2% paraformaldehyde and 15% picric acid) at 5, 8, and 12 W. The eyes were rapidly enucleated and fixed in 3% glutaraldehyde solution (3% glutaraldehyde, 9% formaldehyde, 37.5% ethanol, and 12.5% acetic acid in distilled water) for 2 h. Paraffin-embedded retinal cross sections of 7μm thickness were cut through the optic nerve and examined with hematoxylin and eosin staining. The number of neurons in the GCL and the thickness of the IRL (from the internal limiting membrane to the inner boundary of outer nuclear layer) were measured, as we previously reported^83^.

### Retrograde labeling

Retrograde labeling of RGCs was conducted as we previously described^11,49,53,83^. Briefly, RGCs were retrogradely labeled by injection of FG (Fluorochrome LLC, Denver, CO, USA) dissolved in PBS into the superior colliculus. Ten days after FG injection, these mice were sacrificed and the eyes were enucleated at 8 and 12 W. The retinas were dissected, fixed in 4% paraformaldehyde in PBS for 20 min and flat-mounted on microscope slides for examination under a fluorescence microscope. The number of labeled RGCs was obtained from one central (0.1 mm from the optic disc), one middle (0.8 mm from the optic disc), and one peripheral (1.5 mm from the optic disc) areas (0.04 mm^2^) per quadrant of each retina, and expressed as number per square millimeter^11,49,53,83^.

### Immunohistochemistry

Mice were perfused with ice-cold PBS, followed by Zamboni’s fixative. The eyes were rapidly enucleated, fixed for 2 h in the same solution, and then transferred into a sucrose buffer (30% sucrose in a 0.1 M phosphate buffer) for cryoprotection for 24 h. The eyes were embedded in Tissue-Tek OCT Compound (Sakura Finetechnical, Tokyo, Japan) and frozen. Retinal sections of 10μm thickness were cut on a cryostat at −20 °C and examined by immunostaining using antibodies against GLAST (1:1000; Rb-Af660; Frontier Institute, Hokkaido, Japan), GS (1:1000; MAB302; Merck Millipore), RBPMS (1:1000; ABN1376; Merck Millipore, Burlington, MA, USA), Calretinin (1:1000; 66496–1-Ig; Proteintech, Chicago, IL, USA), Calbindin (1:1000; 14479–1-AP; Proteintech), PKC (1:1000; 21991–1-AP; Proteintech), GSH (1:200; J100; Signature Immunologics, Salt Lake, UT, USA), 4-HNE (1:1000; MHN100P; Japan Institute for the Control of Aging, Shizuoka, Japan), and LC3B (1:1000; L7543; Sigma-Aldrich, St. Louis, MO, USA). ImageJ (http://imagej.nih.gov/ij/; provided in the public domain by the National Institutes of Health, Bethesda, MD, USA) was used for analyzing the intensity of these staining^11,84^.

### Statistics

Data are expressed as mean ± S.E.M. When statistical analyses were performed, the one-way ANOVA followed by the Tukey’s post hoc test was used for multiple comparisons. Results were considered statistically significant when *P* < 0.05. JMP version 12.2.0 (SAS Institute Inc., Cary, NC, USA) was used for statistical analyses. For each experiment, more than three animals were randomly chosen and used for analysis. No animals met the exclusion criteria (for example, premature death, surgical failure, etc.) The investigators were blinded with respect to the treatment of mice.
